# Self-Motion Holds a Special Status in Visual Processing

**DOI:** 10.1371/journal.pone.0024347

**Published:** 2011-10-05

**Authors:** Roy Salomon, Sarit Szpiro-Grinberg, Dominique Lamy

**Affiliations:** 1 Department of Psychology, Tel Aviv University, Tel Aviv, Israel; 2 Department of Psychology, New York University, New York, New York, United States of America; Royal Holloway, University of London, United Kingdom

## Abstract

Agency plays an important role in self-recognition from motion. Here, we investigated whether our own movements benefit from preferential processing even when the task is unrelated to self-recognition, and does not involve agency judgments. Participants searched for a moving target defined by its known shape among moving distractors, while continuously moving the computer mouse with one hand. They thereby controlled the motion of one item, which was randomly either the target or any of the distractors, while the other items followed pre-recorded motion pathways. Performance was more accurate and less prone to degradation as set size increased when the target was the self-controlled item. An additional experiment confirmed that participant-controlled motion was not physically more salient than motion recorded offline. We found no evidence that self-controlled items captured attention. Taken together, these results suggest that visual events are perceived more accurately when they are the consequences of our actions, even when self-motion is task irrelevant.

## Introduction

You are gazing into a glass window reflecting the people walking by. You are searching for a specific target (say, a friend) in the window reflection. While searching you continue walking by the window as do the people around you. Would your own reflection in the window look more salient to you relative to the reflections of others, based only on the fact that it makes the same movements as you? If you wished to determine beyond any doubt whether a candidate figure is indeed your own reflection, you would typically make a conspicuous movement to verify that the figure moves accordingly. Yet, if you just continued searching for your friend, would the reflection of your movements stand out against the background of the moving crowd, despite being irrelevant to the task you are engaged in?

If so, this situation would exemplify that our movements can be used to assist us in conditions in which self-recognition is not trivial. A fundamental aspect of our sense of self is agency, which refers to our ability to exert willed control over the movements of our bodies (agency). The sense of agency relies on both efferent information, that is, centrally defined motor plans that provide information about our intended movements, and afferent information, that is, various sensory inputs (visual, tactile, and proprioceptive) monitoring the execution of these motor plans. The interplay between these sources of information allows us to continuously distinguish between the consequences of our actions and the consequences of actions that are unrelated to our own, and thereby to distinguish our own bodies and movements from those of others [1], [2], [3].

**Figure 1:**
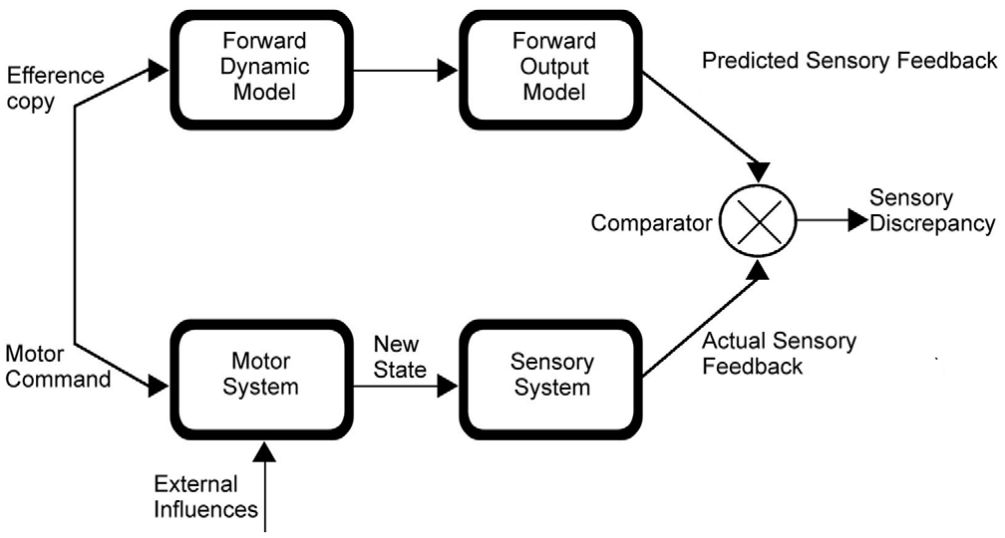
Schematic representation of the comparator model. Each motor command produces an efferent copy which is convolved into a forward model predicting the sensory consequences of the motion. Afferent sensory systems provide real-time information regarding the actual state of the system and these are compared with the predicted states of the forward model.

The predominant account for our sense of agency is often referred to as the “forward model account” [4], [5], [6]. It posits that an “efference copy” is created when we make an intentional movement [6], [7], [8] - see figure 1 - and that “copies” of the planned motor actions are compared to the afferent signals arising from sensory inputs [6]. When the efferent and afferent signals are congruent, we attribute the movement to ourselves (agency). When they mismatch, we deduce that the movement does not arise from our own will (as for passive movements). The efference copy allows us to prepare for the predicted consequences of our movements and monitor the progress of their execution. The model implies that we possess precise information regarding our intended movements and their consequences. However, the processes by which the comparator detects matches and discrepancies between intended movements (efferent information) and their sensory consequences (afferent information) are yet under investigation. In particular, it is unclear how attention interacts with the comparison process.

In line with the model, investigations of the role of efference copy in self-recognition from motion suggest that sensory information is indeed better represented when it is a consequence of willed action, presumably because the efference copy provides an additional source of information. For instance, Tsakiris and colleagues (2005, see also [9]) had participants decide whether a visual stimulus represented their own moving hand or someone else's, while all visual cues were equated between the two conditions. Participants were more accurate when they actively initiated their movement than when their hands executed the same movement, but passively. This finding suggests that “central signals are highly accurate in detecting the appropriate afferent signals that pertain to one's self” [10 p. 402]. Accordingly, one might predict that an object should be more salient when its motion corresponds to one's own movements.

However, the results from a different line of research suggest that when self-recognition is not required, fairly large discrepancies between one's movement and its visual consequences go unnoticed [11], [12]. In a classic experiment, Nielsen had participants draw a straight line, but unbeknownst to the participants, an angular deviation from the participants' own movements was introduced using a mirror. The participants corrected for the deviation by moving their unseen hands in the opposite direction, yet remained unaware of this correction [12], [see also13], [14]. While such findings suggest that the outcomes of the comparison process are not automatically brought to our attention or consciousness, they do suggest that extensive processing of afferent and efferent signals took place since observers adjusted their movements with high precision to compensate for the discrepancy from their perceived outcomes. In addition, these studies did not involve a comparison between processing of stimuli that match the observers' willed motion and processing of stimuli that do not.

Thus, we cannot determine, based on the extant literature, whether or not an object enjoys privileged processing when its motion follows our willed movements relative to when it follows an unrelated path, when monitoring the match between the two motions is irrelevant to the task at hand.

The objective of the present study was to investigate this issue. To do that, we employed a novel adaptation of the irrelevant-singleton paradigm pioneered by Yantis and colleagues [e.g.,15] to study attentional capture by salient stimuli. In this paradigm, participants search for a pre-specified letter among a variable number of non-target letters, and on each trial, a salient item (e.g., an abruptly onset item or an item with a unique color) is also present. The positions of the target and salient distractor are uncorrelated. The prediction is that if the salient item enjoys processing priority, then it should be easier to respond to the target when it happens to coincide with the salient distractor than when it does not, which should be reflected in shallower search slopes in the former relative to the latter condition. The results [15] confirmed this prediction when the critical item was abruptly onset but not when it was a color singleton, suggesting that the former stimulus type mandatorily captures attention while the latter does not.

Here, participants searched through a display of moving items and controlled the motion of one of the search items. The self-controlled item was as likely as any of the other items to be the target. We predicted that if an object is better processed when its motion is controlled by the observer, search should be more efficient when participants happen to control the target's motion than when they happen to control a distractor's motion.

**Figure 2:**
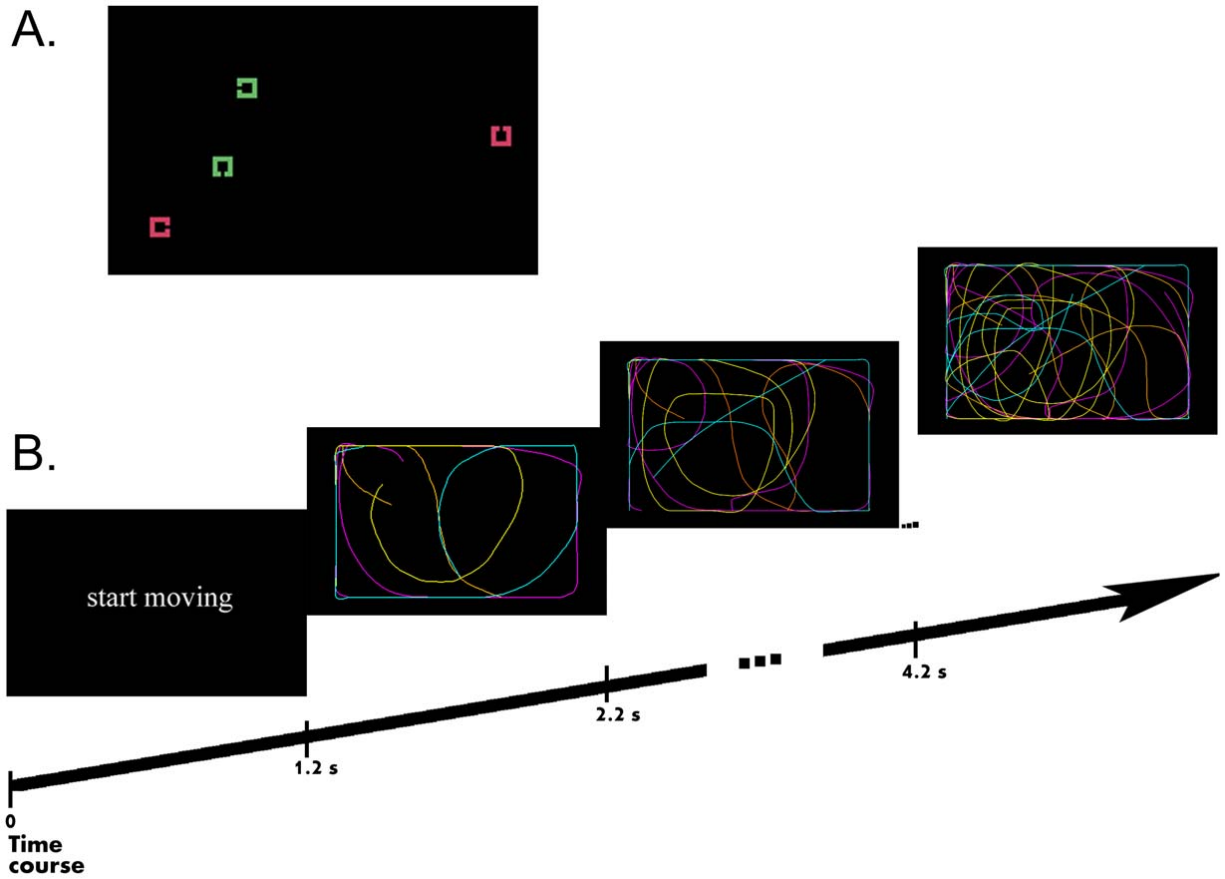
Schematic representation of the sequence of events during a sample trial. A. Sample set-size 4 display. The participants' task was to identify the color of a target stimulus defined for each participant by the position of the gap in its outline. In the example, the target is defined as the square with a gap on the left side. The stimuli in this figure are larger than the actual stimuli. All stimuli were in constant motion throughout the trial. B. Color lines illustrate the motion paths of the stimuli (and were therefore not present in the displays). Note multiple regions of convergence of motion paths, making the visual search task difficult. The yellow line represents the path of the motion controlled by the participant. The other lines represent pre-recorded motion paths.

Participants were instructed to search for a target defined by its shape among a variable number of distractors (either 3 or 5). They responded to the color of the target with one hand while making a continuous movement holding the computer mouse with their other hand. All stimuli were moving during the search, but one was controlled by the participant's movement (see Figure 2). The motion paths of the remaining stimuli were controlled by prerecorded movement files. The self-controlled stimulus was as likely as any other stimulus to be the target. Thus, participants had no incentive to search for it. Search slopes are typically used to assess search efficiency by measuring the additional performance cost incurred with each distractor added to the display. Null search slopes indicate that the target is highly salient, as the target is responded to equally easily whatever the number of distractors. Large positive search slopes indicate that search is inefficient and that the target enjoys little or no processing advantage over distractors. If self-controlled movements have a special status in visual processing even when agency is utterly irrelevant to the task at hand, then search slopes should be flatter when the target happens to be the self-controlled item than when it is not.

As the critical aspect of our manipulation was the match between the participant's hand motion and the trajectory of one display item, it was important that the participants be exposed to the motion trajectories long enough for these trajectories to effectively differ from one another. Therefore, we had to ensure that search be difficult enough to require more than a few hundreds of milliseconds to complete. We took two main steps to achieve this purpose. First, target and distractors were very similar on the search-critical dimension (shape): thus, the target shape did not pop out and its identification required focused attention [e.g., 16]. Second, the two possible colors present in the display were also similar to each other. This was done in order to prevent the participants from using an alternative search strategy: If color was easier to discriminate than shape, then participants might segregate the display into two color groups and search for the target shape through one color group only (say the red group): they would answer “red” if a target was found in this group and “green” otherwise, without having to search through the green group because, as a target was present on each trial, if it was not red it was necessarily green^1^ [e.g., 17]. A pilot study indicated that when the search task was difficult enough to allow for the required exposure time (mean RTs above 2 sec), it was associated with highly variable RTs and yielded average accuracy rates of ∼75%. Thus, accuracy was the relevant dependent measure in our study (RT data are nonetheless reported in supplementary information S1 and Table S1).

## Experiment 1

### Results and Discussion

Two participants were excluded from the statistical analyses because during the post-experiment interview, they reported that they had purposefully searched for the item the motion of which they controlled.

**Figure 3:**
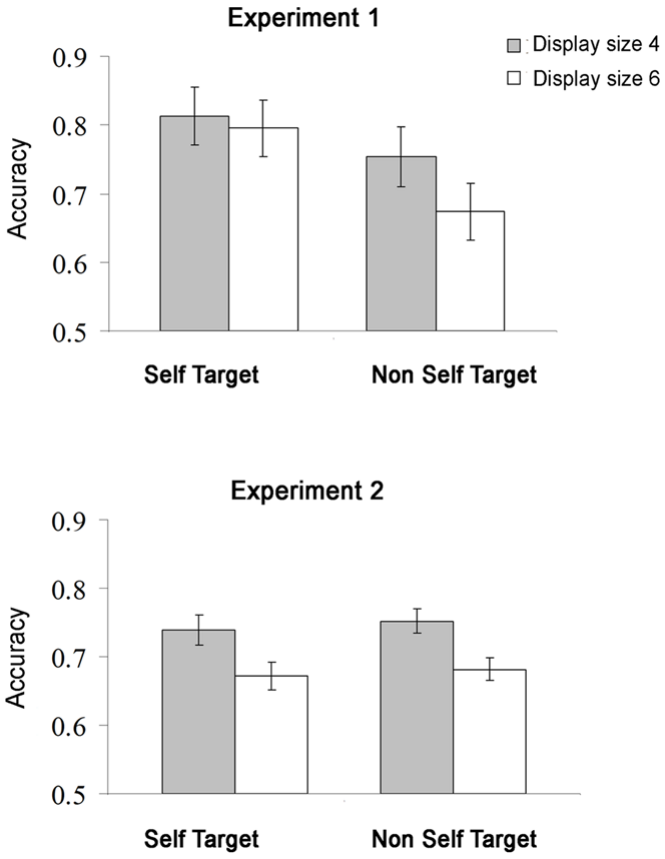
Performance accuracy ratios for each experiment (1 and 2), by target type (self vs. non-self) and set size (4 vs. 6). Experiment 1 (top): Participants were more accurate when the target was the item they controlled than when it was not. Adding distractors impaired accuracy only when the target was not controlled by the participant. Experiment 2 (bottom), in which participants viewed the same displays as in Experiment 1 but did not control the motion of any of the stimuli. There was no advantage for targets that had been controlled by the participant in Experiment 1, thus removing any concern that physical differences between self and non-self motion might underlie the findings from Experiment 1. Error bars represent SEM.

A repeated measures analysis of variance (ANOVA) with target type (self vs. non-self) and set size (4 vs. 6) was conducted on the error rates of the remaining 11 participants. Mean accuracy data are presented in Figure 3A.

Mean accuracy was 75.9%. Performance was more accurate for self than for non-self targets, M = 80.4%, SD = 12.9% vs. M = 71.3%, SD = 12.8%, respectively, F(1,10) = 33.92, p<.0002, Cohen's d = .70. The main effect of set size was also significant, F(1,10) = 8.82, p<.02, Cohen's d = .36, with higher accuracy for set size 4 vs. 6 (M = 78.3%, SD = 13.6% vs. M = 73.4%, SD = 13.3%, respectively), and so was the interaction between the two variables, F(1,10) = 7.53, p = .02. Follow-up comparisons showed that increasing the set size impaired search when the target was not controlled by the participant, with a 7.9% decrement in accuracy in the 6- relative to 4-item displays F(1,10) = 20.19, p<.002. In contrast, search performance was independent of set size when the target motion was controlled by the participant, with only a non-significant 1.8% decrease in performance, F<1.

An additional ANOVA with trial type (self vs. non-self target), practice (first vs. second block of trials) and set size (4 vs.6) showed no interaction involving trial type and practice, Fs<1 (only the main effect of practice and the interaction between practice and set size were significant, ps<0.05). Thus, practice had no differential effect when the target was self-controlled relative to when it was not.

Finally, although the participants were trained to produce movements according to the same rules as those produced offline, we examined whether self-motion and control motion vectors might differ in some systematic way. No difference between average self- vs. control motion was found in velocity (M = 36.64, SD = 10.86 vs. M = 34.5, SD = 9.25, respectively, p>0.05), curvature, (M = 0.17, SD = 0.07 vs. M = 0.16, SD = 0.06, respectively, p>0.05) or acceleration (M = 0.085, SD = 0.02 vs. M = 0.105, SD = 0.09, respectively, p>0.05).

It remains possible, however, that differences in motion characteristics that cannot be detected based on average data might nonetheless have occurred. In particular, participants may have made distinctive movements only as soon as they realized that the target was self-controlled. To evaluate this possibility, we narrowed the comparisons between motion characteristics of self- vs. non-self targets to the time window preceding the participants' response by 200–400 ms. No differences were found in any of the motion parameters (velocity, curvature or acceleration, all p>0.25). As an additional test, we examined the time window preceding the participants' response by 500–700. Again, no differences in were found (all ps>0.4).

During the post-experiment interview, all participants (except the two participants who were excluded from the analysis) reported that they were generally unsure which item they were controlling because they did not pay attention to it. In order to determine whether participants were overall aware of the performance advantage of the self targets, we examined the correlations between the set size advantage in accuracy for self relative to non-self targets (i.e., slope for nonself target minus slope of self target) and participants' subjective reports. The search slopes ranged between 7% and 18% for self-targets, target perceived salience ranged between 1 and 7 (M = 4, SD =  2) and perceived ease of finding the self target ranged between 2 and 7 (M = 4.9, SD = 1.75). Neither the correlation between slope and self target perceived salience nor between slope and perceived ease of finding the self target approached significance, r = 0.18, t<1 and r = −0.3, t(10) = 1.14, respectively. In addition, none of the participants (except the two participants who were excluded from the analysis) reported adopting, be it even partially, the strategy of looking for the item they were controlling. Their subjective report was that moving their hand had become automatic after practice and they no longer thought about it during the difficult search.

The results show that when the target motion happened to be controlled by the participant, performance was more accurate and did not decrease as the number of distractors in the search display increased. However, although instructions and training were aimed at minimizing the probability that the movements initiated by the participants might be perceptually different from the movements controlled by pre-recorded motion files, and although no significant differences between these emerged on velocity, curvature, or acceleration, we cannot exclude the possibility that other motion parameters that we failed to consider may have made self-motion more salient in ways that escaped the participants' awareness. Experiment 2 was designed to test whether such potential perceptual differences between self and non-self motion items might indeed account for the performance advantage observed for self versus non-self targets.

In this experiment, each new participant was yoked to a participant in Experiment 1. That is, the participant viewed exactly the same displays as the participant of Experiment 1 to whom she or he was yoked. Thus, the trials were identical to those used in Experiment 1. The only difference was that although the participants in Experiment 2 were moving their right hands just as the participants in Experiment 1 had, their movement did not control any of the items in the search display. If the findings from Experiment 1 resulted from the physical salience of the movements initiated by the participants, then in Experiment 2 we should also observe improved search performance when the target happened to be an item the motion of which had been controlled by the participant in Experiment 1. By contrast, if search performance does not differ whether the motion of target had or had not been controlled by the participants in Experiment 1, then we can safely conclude from the findings of Experiment 1 that self-initiated motion indeed has a special status for visual processing, even when this motion is not physically more salient than motion initiated by someone else.

## Experiment 2

### Results and Discussion

The same ANOVA was conducted as in Experiment 1. Mean accuracy data are presented in Figure 3B. Mean accuracy was 71.1%. Participants were more accurate in the set size 4 (M = 74.5%, SD = 6.2%) than in the set size 6 (M = 67.7%, SD = 5.7%) condition, F(1,10) = 28.84, p<.0003. The effect of target type and its interaction with set size were not significant, Fs<1. Thus, search performance was equally impaired by increasing set size, whether or not the motion of the target had been controlled by the participant in the main experiment, 6.7% vs. 7.0%, respectively.

These findings rule out the possibility that the findings from Experiment 1 might have resulted from perceptual differences between self and non-self motion.

## General Discussion

The findings of the present study suggest that an object that moves in spatio-temporal congruence with our own willed movement enjoys a special status in visual search even when it is task irrelevant. Crucially, the results of the control experiment confirmed that this performance advantage did not result from potential physical differences between the motions of self and non-self items but from the fact that participants controlled the target's motion. Search performance was more accurate and less impaired, if at all, by the addition of distractors to the search display, when the participants controlled the target's motion than when they controlled the motion of a distractor.

Accuracy is often used as an alternative measure to reaction times for determining search efficiency when the displays are viewed under data-limited conditions [18]. In such studies, the search display is typically presented very briefly and followed by a mask. Participants are required to find the target, that is, to extract the task-relevant information, before the search display is replaced by the mask. The time available for responding is typically unlimited. Accuracy rates, which are the dependent variable of interest, are typically low (circa 75%) and RTs are not analyzed. Differences in accuracy across conditions are thought to reflect differences in how well information can be extracted from the display before it becomes unavailable.

In our study, accuracy rates were low, yet the conditions that prevailed were not typical of data-limited conditions: While the displays remained in view fairly long (6000 ms), their representations had to be constantly updated, because all the items making up the search array continually moved, throughout each trial. Thus, the low accuracy rates we observed could result from two different types of difficulty. On the one hand, the time allotted to finding the target may not have sufficed, thereby leading to a large proportion of trials in which the target could not be found. On this account, the shallower slopes observed for self-targets would indicate that the quality of the perceptual representation is higher for objects that move according to our own willed movements than for objects that move independently. This in turn would allow us to infer that these objects may enjoy higher attentional priority due to their higher perceptual salience.

On the other hand, however, because targets and non-targets moved constantly across the display and their paths repeatedly crossed one another, tracking of the target was quite difficult, such that participants may often have “lost” the target. That is, participants may have found it within the allotted time, but then they may have confused it with a distractor by the time they had to respond to the target color, and more often so when the number of distractors was high than when it was low. According to this interpretation, not the representations of the task-relevant properties (here, shape and color) are more salient in a self- vs. non self-motion target, but the representation of its motion, which can therefore be tracked more easily after it has been found.

In order to test these alternative interpretations against each other, we looked at the proportion of trials in which the target could not be found within the allotted time. We found it to be quite low (around the 3% of all trials, that is, 14% of all error trials – which is not surprising given the fact that average RTs approximated 2,000 ms). This finding suggests that the main difficulty of the task was not to find the target but once it was found, to correctly report its color. Thus, successful performance in this task was contingent on the observer's ability to avoid “noise” caused by the other distractors and the constant motion. It should be noted, however, that our data do not speak to whether or not objects the motion of which we control enjoy higher attentional priority. On the one hand, the conditions that prevailed in our task were not data limited in the sense that the relevant data could be extracted within the allotted time. Differences in accuracy between self and non-self targets therefore did not reflect differences in the quality of perceptual processing. On the other hand, however, the high error rates did not allow us to rely on RT data, which indeed proved to be very noisy^2^, thus precluding the possibility to infer attentional priority from differential search slopes on the RT measure.

Taken together, the present findings suggest that once attention focuses on an object the motion of which follows our own willed movements it is easier to track it and to identify its task-relevant properties relative to an object that moves following an unrelated path. These findings are consistent with the notion that efferent signals that are associated with self-initiated action provide detailed temporal and kinetic information pertaining to the consequences of self-initiated action [9], [19]. Such efferent information was useful once the target had been detected, because it allowed the participant to extract the color of the target while it was moving, with less impairment from the noise created by distractors that were similar to the target and constantly crossed its path. Accordingly, relative to a non-self-target, a self-target appeared to be shielded from additional noise and participants were more accurate in reporting its color.

Previous studies have shown that the perceptual consequences of self-generated actions are attenuated, and that such attenuation may even serve as an index of agency (e.g., Blakemore et al., 1999; Weiskrantz, Elliot, & Darlington, 1971; Wolpert et al., 2000). For instance, relying on the observation that we cannot tickle ourselves, Blakemore et al. used a robotic arm to introduce spatial and temporal deviations between participants' planned actions and their sensory outcomes. The participants' task was to judge the “ticklishness” of the sensory stimulation. The larger the discrepancy between the self-produced action and the tactile outcome, the more ticklish the tactile sensation was perceived to be.

Similarly, in the visual domain, such “canceling out” of the effects of self-generated movements is necessary for stabilization of visual perception during eye, head, and body movements [20]. In these cases, the efferent copy allows one to separate the effects of self-generated motion from externally generated effects arising from the environment.

Accordingly, if a mechanism similar to suppression for self-generated somato-sensory stimulation exists for visual stimulation, we would expect an object the motion of which we control to be perceptually less salient and thus to be less likely to draw our attention than an object that follows a different motion path. Yet, in the present study, the perceptual consequences of self-initiated hand motion were not attenuated: Instead, they were found to hold a privileged status, which suggests that they may serve a different purpose.

One may speculate that implicit monitoring of hand motion (which unlike central body motion or leg motion is often visible) might be important for the comparison process that allows one to detect discrepancies between intended and executed motion. Consistent with this speculation, several studies have shown that visual information near or on the hand can substantially affect the sense of embodiment [21], [22] and motor judgments [12], [13]. Furthermore, there is evidence that peripersonal space near the hands is represented in specific brain regions regardless of the location of the hand in space [23]. Thus, attenuation of the perceptual consequences of self-generated motion may not always be desirable, and notably in the case of self-generated hand motion.

Recently, a distinction was proposed between two levels of agency: a feeling of agency which is automatic and implicit; and a second-order explicit judgment of agency, which is a reflective process [24], [25]. Support for this distinction was recently shown in a patient with anosognosia for hemiplegia, who made online motor corrections for angular perturbations of the consequences of his movements with his healthy hand, while showing no awareness of these perturbations [16]. Our results are consistent with this theoretical distinction in suggesting that self-generated movements hold a privileged position in visual perception even when they are task irrelevant and therefore not explicitly monitored.

The findings from the present study considerably broaden the range of situations in which efferent information may help us monitor the outcomes of self-initiated actions. They show that visual events are perceived more accurately when they are the consequences of our actions. Moreover, such improved performance does not seem to be either mediated by or contingent on explicit self-attribution of our actions consequences: The reports gathered during the post-experimental interview revealed that participants were largely unaware of which item had been under their control during search.

## Methods

### Experiment 1

#### Participants

Thirteen Tel-Aviv University undergraduate students (4 males, aged 20 to 26) participated for course credit. All were right-handed, reported normal or corrected-to-normal visual acuity and normal color vision. This experiment was specifically approved by the ethics committee at Tel Aviv University and was conducted according to the principles of the Declaration of Helsinki. Written informed consent was obtained from all participants in accordance with the guidelines of the Tel Aviv University ethics committee.

#### Stimuli

Stimuli were displayed against a black background, inside an invisible black rectangle subtending 89×112 mm and located in the center of the computer monitor. Each search display consisted of either four or six outlined squares subtending 6.4 mm in side, with a 1-mm gap on one of each square's four sides (up/right/down/left). Each square was either light red (RGB = 145,140,125) or light green (RGB = 125,145,131). These values yielded colors which were not easily discriminable (see supplementary information S2). Each search display consisted of an equal number of green and red items. In the display-size 4 condition, each square presented a gap on a different side. In the display-size 6 condition, the gap appeared on the same side for two pairs of squares while the remaining two squares (one of which was the target) had a unique gap side (Figure 2). The instruction display illustrating the target consisted of one white square similar to the squares appearing in the search display and presented in the middle of the screen.

#### Motion files

All stimuli, except for the self-controlled stimuli, moved following paths randomly selected from a large database of motion files (6,700 and 6887 motion files for set sizes 4 and 6, respectively) created by different participants in a pilot study. The pilot study was designed to produce motion files recorded from participants who performed the same search task as the participants in the main experiment, and under similar conditions. This was important in order to ensure that motion would be highly similar in the self and non-self conditions. During each trial the motion of one stimulus followed the motion of the mouse controlled by the participant in real time, while the motions of the distractors (either three or five) were randomly selected from the motion files pool.

#### Procedure

Participants were told that the experiment measured their ability to perform two unrelated tasks simultaneously. The primary task was a visual search task and the secondary was to move their right hands continuously. The instruction display showed the target template for the search task: The target was defined as the square with a gap on the same side as that on the square presented in the instruction display, and remained the same throughout the experiment. The participants had to report the color of the target, red or green, by pressing designated keys with their left hands. They were instructed to respond as accurately as possible while maximizing speed. With their right hands the participants controlled a mouse, which they had to move continuously throughout the trial duration.

It was crucial to ensure that the participants' movements should be physically similar to those in the control motion files, in order to prevent a situation where the self-controlled item might be salient due atypical motion characteristics. Hence, during the practice session we trained participants to produce movements that conformed to specific constraints. Participants were told that the movement should cover all four quadrants of the display, be continuous, start with the presentation of the “start moving” instruction and be neither too fast nor too slow. The participants were also specifically instructed that their hand should move only within the borders of the mouse pad. The experimenter monitored the participant's movement during practice until she or he was convinced that the movements had the required characteristics. Such monitoring was done only during training and participants usually became proficient with these motion rules within the first practice block.

The participants were informed that the motion of one of the display stimuli on each trial was controlled by the mouse, but they were also explicitly instructed that this stimulus was no more likely than any other stimulus in the display to be the target and so they should not attempt to look for it. To avoid that the self-controlled stimulus be stationary and therefore conspicuous among the moving items at the trial onset, each trial began with a “start moving” written instruction, that cued the participants to begin their movements before display onset (and therefore, with no visual feedback). After 1200 ms, the target display appeared with targets and distractors already in motion, including the self-controlled item. The target display was presented until response or until 6 seconds had elapsed. The stimuli did not rotate during motion.

At the end of the experiment, the participants were interviewed in order (1) to assess the extent to which the participants thought that the item they had controlled appeared to be salient (on a scale of 1 to 10) or easier to find (on a scale from 1 to 10) and (2) to screen out participants who might have used the strategy of looking for the self-controlled item during search. Then, the participants were debriefed by the experimenter.

#### Design

The self-controlled stimulus was as likely as any other stimulus to be the target (it was the target on 25% of the trials for set size four and in 16.6% of the trials for set size six). All conditions of set size and target color were equally probable. Conditions of control over the target motion (self-controlled vs. non-controlled; henceforth, self vs. non-self motion) set size, and target color were randomly mixed within each block of trials. Conditions of set size were run in separate blocks of trials. The side of the gap on the target square remained fixed throughout the experiment for each participant and was randomly assigned to each participant.

The experiment began with four blocks of practice trials, with set size 4 (7 trials) and set size 6 (12 trials) in an ABAB order. This order was not counterbalanced because performing the set-size 6 condition proved to be extremely difficult if it had not been preceded by practice on the set-size 4 condition, in line with the findings from previous studies [26] showing that learning is easier when easy exemplars are presented before difficult ones. In all practice blocks, the target stimulus was the same as in the experimental blocks. There were four experimental blocks (2 blocks per set size in an ABAB order), with each block including 162 trials.

### Experiment 2

#### Participants

Eleven Tel-Aviv University undergraduate students (2 males aged 19 to 34) participated in the experiment for course credit. All were right-handed, reported normal or corrected-to-normal visual acuity and normal color vision. We followed the same procedure as in Experiment 1 to comply with ethical requirements.

#### Stimuli and procedure

The stimuli and procedure were identical to Experiment 1 except for the following changes. The durations of the self-controlled motions initiated by the participants in Experiment 1 were determined by their reaction times. Thus, motion files in Experiment 2 had to be either truncated or prolonged. When the participant in Experiment 2 responded faster than the participant from Experiment 1 to whom she or he was yoked, the motion file was simply stopped as soon as the participant responded. When the participant in Experiment 2 responded slower, the motion file was run backwards after it had run to its end, until the participant responded or until 6 seconds had elapsed. This allowed the motion to remain smooth, with no interruption at the time point when the actual motion had ended in Experiment 1. As in Experiment 1, participants were informed that sometimes the motion of an item would be controlled by their hand movement, but that this item would be as likely as any other to be the target. This false information was rectified during debriefing.

## Supporting Information

Table S1 Reaction Times for all experimental conditions. Mean reaction times (RTs) and standard deviations for Experiments 1 and 2 by conditions of target type and set size.
(DOCX)

Supplementary Information S1 Analysis of Reaction Times.
(DOCX)

Supplementary Information S2 Color and Shape Discrimination control Experiment.
(DOCX)
