# Supplementary material for: Self-Motion Holds a Special Status in Visual Processing
Source: PLoS One. 2011 Oct 5;6(10):e24347. doi: 10.1371/journal.pone.0024347 (PMC3187743; doi:10.1371/journal.pone.0024347)
Supplement: Table S1 — Reaction Times for all experimental conditions. Mean reaction times (RTs) and standard deviations for Experiments 1 and 2 by conditions of target type and set size. (DOCX) [file pone.0024347.s001.docx]

**Table S1:** Mean reaction times (RTs) and standard deviations for Experiments 1 and 2 by conditions of target type and set size.

|  | **Self** | | | | **Non-self** | | | |
| --- | --- | --- | --- | --- | --- | --- | --- | --- |
|  | *Size 4* | | Size 6 | | Size 4 | | Size 6 | |
|  | *mean RT* | *Std* | *mean RT* | *Std* | *mean RT* | *Std* | *mean RT* | *Std* |
| **Experiment 1** | 2309 | 416 | 2387 | 651 | 2660 | 363 | 2547 | 457 |
| **Experiment 2** | 2755 | 565 | 2644 | 452 | 2679 | 346 | 2679 | 532 |
