# Supplementary material for: Self-Motion Holds a Special Status in Visual Processing
Source: PLoS One. 2011 Oct 5;6(10):e24347. doi: 10.1371/journal.pone.0024347 (PMC3187743; doi:10.1371/journal.pone.0024347)
Supplement: Supplementary Information S1 — Analysis of Reaction Times. (DOCX) [file pone.0024347.s002.docx]

**S1. Analysis of Reaction Times.**

An ANOVA with target type (self vs. non-self) and set size (4 vs. 6) as within-subject factors was conducted on the RT data (excluding error trials). In Experiment 1, in which the participants controlled the motion of one of the search items, only the main effect of target type was significant, with faster RTs for self- relative to non-self-targets, F(1,10)=5.94, p<.04. The main effect of set size was not significant, F < 1 and neither was the interaction between the two variables, F(1, 10) = 1.37, p > 0.2. In Experiment 2 in which the participants did not control the motion of any of the search items but were yoked to participants of Experiment 1, the main effects and interaction were not significant, all Fs < 1.
