# Supplementary material for: Self-Motion Holds a Special Status in Visual Processing
Source: PLoS One. 2011 Oct 5;6(10):e24347. doi: 10.1371/journal.pone.0024347 (PMC3187743; doi:10.1371/journal.pone.0024347)
Supplement: Supplementary Information S2 — Color and Shape Discrimination control Experiment. (DOCX) [file pone.0024347.s003.docx]

**S2: Color and Shape Discrimination control Experiment**

We ran a control experiment designed (1) to ascertain that color discrimination was indeed more difficult than shape discrimination and (2) that color discrimination was not liminal. Eight new participants were presented with a centrally positioned stimulus that appeared for 200 msec and could vary in color and in shape (in the same way as did the stimuli in the main experiments). In the color condition, they had to respond to its color (either red or green, which were the same "faint" colors as in the original experiment) and in the shape condition, they had to respond to its shape (one of the 4 possible shapes). Mean RTs were 710 ms (STD=72) vs. 504 ms (STD=53.4) in color vs. shape condition, respectively and mean accuracy was 0.83 (STD=0.08) vs. 0.98 (STD=0.02) in color vs. shape condition, respectively. These results suggest that color discrimination was indeed more difficult than shape discrimination and that color discrimination was not liminal.
